# Study of mucin turnover in the small intestine by *in vivo* labeling

**DOI:** 10.1038/s41598-018-24148-x

**Published:** 2018-04-10

**Authors:** Hannah Schneider, Thaher Pelaseyed, Frida Svensson, Malin E. V. Johansson

**Affiliations:** Department of Medical Biochemistry and Cell Biology, University of Gothenburg, Gothenburg, Sweden

## Abstract

Mucins are highly glycosylated proteins which protect the epithelium. In the small intestine, the goblet cell-secreted Muc2 mucin constitutes the main component of the loose mucus layer that traps luminal material. The transmembrane mucin Muc17 forms part of the carbohydrate-rich glycocalyx covering intestinal epithelial cells. Our study aimed at investigating the turnover of these mucins in the small intestine by using *in vivo* labeling of *O*-glycans with *N*-azidoacetylgalactosamine. Mice were injected intraperitoneally and sacrificed every hour up to 12 hours and at 24 hours. Samples were fixed with preservation of the mucus layer and stained for Muc2 and Muc17. Turnover of Muc2 was slower in goblet cells of the crypts compared to goblet cells along the villi. Muc17 showed stable expression over time at the plasma membrane on villi tips, in crypts and at crypt openings. In conclusion, we have identified different subtypes of goblet cells based on their rate of mucin biosynthesis and secretion. In order to protect the intestinal epithelium from chemical and bacterial hazards, fast and frequent renewal of the secreted mucus layer in the villi area is combined with massive secretion of stored Muc2 from goblet cells in the upper crypt.

## Introduction

The small intestinal epithelium is constantly aiming to balance effective nutritional uptake with minimal damage due to exposure to ingested, secreted and resident agents. Detrimental challenges, including high concentrations of acid, digestive enzymes and bile, dominate in the upper small intestine. More distally, the increasing number of commensal bacteria poses the predominant threat. The intestinal epithelium is a single-cell layer with tightly connected cells that form a barrier towards the extracellular environment with selective transport and abilities to sense and respond to luminal antigens. Secreted mucins constitute a significant part of the intestinal epithelial protection by forming a gel to maintain surface hydration and reduce mechanical stress. The small intestinal mucus layer acts as a diffusion barrier that can establish pH and antimicrobial gradients which in turn protect the underlying cell surfaces^1,2^. In order to maintain its protective function, the mucus undergoes continuous renewal by secretion. Intestinal secreted mucus is a complex network built around multimers of the highly *O-*glycosylated MUC2 mucin^3,4^, which is produced by specialized cells, the goblet cells (GCs). Early during biosynthesis in the endoplasmic reticulum, *N*-glycosylated, disulfide-linked dimers of MUC2 are formed, which are then transferred to the Golgi apparatus where *O*-linked glycans are attached to serine and threonine residues within MUC2’s long central proline threonine serine-rich (PTS) domain. Additional disulfide bonds generate multimers of MUC2, which are packed and stored in mucin granules for later secretion into the lumen^5^. Mucin secretion is most likely a continuous process with an intrinsic basal rate, which upon specific danger signals can be increased to enhance protection.

The small intestinal epithelium is renewed from the adult stem cells and a proliferative cell pool localized in the lower crypt. Differentiation into various cell types is regulated by transcriptional control with differentiated cells migrating upwards from the crypt bottom along the villus axis^6^. Various methods have been utilized to study cell turnover in the small intestine, placing the average time in the range of one to five days depending on methodology^6–8^. Mucus-producing GCs are part of the secretory cell lineage, with mucus-containing cells constituting 4–12% of small intestinal epithelial cells, with the highest proportion in the distal intestine^8^. With the exception of crypt-resident long-lived Paneth cells, all mucus-producing cells differentiate and migrate at the same rate as the remaining epithelial cell types^9^. Although the morphologies of GCs in the crypts and at the villi differ, functional discrepancies in respect to mucus biosynthesis and secretion remain insufficiently understood.

Additional protection for the intestinal epithelium is provided by the carbohydrate-rich glycocalyx, which covers epithelial cells and extends up to one micrometer into the lumen. The glycocalyx is composed of glycoproteins such as transmembrane mucins, which constitute the most glycosylated component. In the human small intestine the most abundant transmembrane mucins are MUC13 and MUC17^10,11^. MUC13 is a short mucin (512 amino acids) with limited reach into the lumen. The considerably longer MUC17 (4,493 amino acids) has an extended, heavily *O*-glycosylated PTS domain, which reaches farthest into the intestinal lumen. Although probably of high importance, knowledge about the function of transmembrane mucins is still limited. Additionally, the literature on transmembrane mucins is elusive due to confusion in terms of available genomic sequences and nomenclature. In humans, the three transmembrane mucins MUC3, MUC12 and MUC17 are clustered on chromosome 7. For a long time, only one of these three transmembrane mucins was mapped in the mouse genome. Initially it was assumed to be Muc3 but was later demonstrated to corresponded to the human orthologue MUC17, resulting in the designation Muc3(17). As all three transmembrane mucins have now been identified in mouse, we call murine MUC17 simply Muc17, thus rejecting the name Muc3(17).

Previous studies have shown that several transmembrane mucins are regulated by PDZ proteins via a C-terminal binding motif. The functionally conserved interaction between MUC17 and PDZ domain containing 1 (PDZK1) stabilizes MUC17 at the apical cell surface and coordinates its trafficking with ion transport in the duodenum^12,13^. Like most transmembrane mucins, MUC17 features an extracellular sea-urchin sperm protein, enterokinase and agrin (SEA) domain located close to its transmembrane region. This SEA domain is autocatalytically cleaved in the endoplasmic reticulum after which the two resulting parts remain linked by non-covalent bonds^14^. This generates a breakpoint, which likely acts as a mechanism to protect cell membranes from mechanical stress by detaching the large glycosylated extracellular part^15^. However, the only studied function of MUC17 so far is its involvement in protection from bacterial invasion using cell lines and a 3D model^16,17^. Turnover of the glycocalyx in general and MUC17 in particular are not yet understood, but it is to be expected that the dynamics of these molecules will impact their role and possible protective function.

By taking advantage of the extensive *O*-glycosylation present in mucins we have investigated the stability and turnover of expelled mucus and the cell-attached glycocalyx. We have previously used *in vivo* labeling by employing *N*-azidoacetylgalactosamine (GalNAz) with subsequent fluorescence detection on fixed samples to study colonic mucus turnover^18^. Here, we have used the same technique to analyze mucus and glycocalyx renewal, in particular Muc2 and Muc17, in the upper mid and distal mouse small intestine. Our data suggest altered biosynthesis rates for secreted mucins, produced by different populations of GCs, while transmembrane mucins in the glycocalyx are stably expressed and display slow turnover.

## Results

### GalNAz labeling of Muc2 as a tool to study small intestinal mucus turnover

Muc2 is secreted by GCs localized in the crypts and along the villi. To study production and renewal of the small intestinal mucus from different cell populations, we took advantage of the highly *O*-glycosylated PTS/mucin domain. During *O*-glycosylation, *N*-acetylgalactosamine (GalNAc) is the first glycan to be attached to the core amino acids threonine and serine. Using an *in vivo* labeling approach the GalNAc analogue GalNAz was incorporated during mucin biosynthesis, followed by visualization with a red fluorophore, added via a Click-iT^TM^ reaction. Due to their heavily glycosylated nature (glycans represent >80% of the total mucin mass) as well as their high abundance, small intestinal mucins will be the predominant components labeled by GalNAz. Although other molecules with fewer GalNAc will also be labeled, these will constitute a minority of the total incorporated GalNAz label. This approach allows us to follow Muc2 biosynthesis and secretion from GCs in different segments of the small intestine.

**Figure 1:**
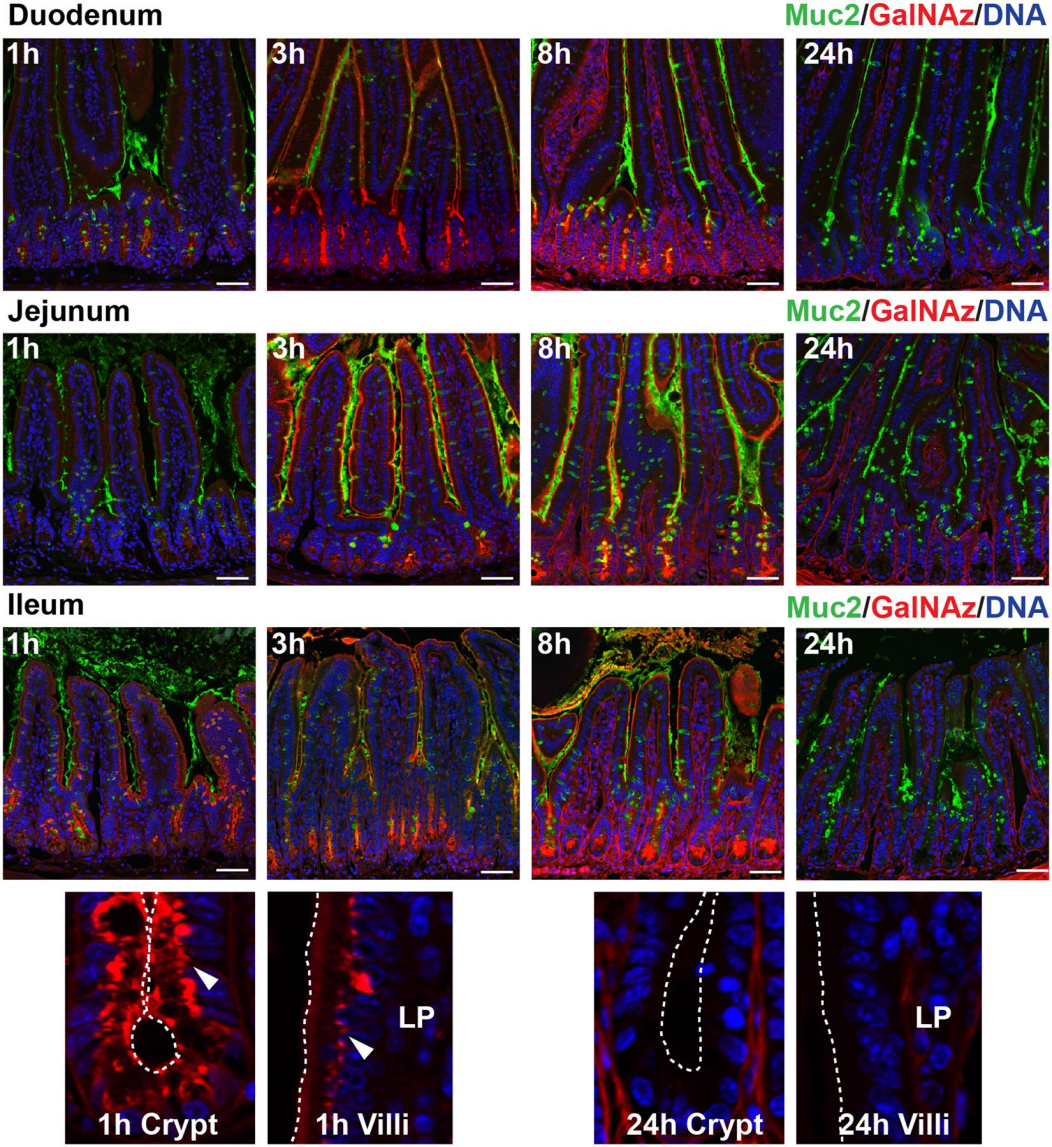
GalNAz labeling of Muc2 is used to analyze small intestinal mucus turnover. Incorporated GalNAz detected by TAMRA (red), immunostaining of Muc2 (green) and DNA stain using Hoechst (blue). Intracellular red staining was first observed at 1 h after injection. Secretion of labeled mucus was seen after 3 h. TAMRA staining was still clearly visible at 8 h but was mostly absent at 24 h (full sets are shown in Supplementary Figs S1–S3). Magnifications of crypt and villi epithelium are shown below after 1 h of labeling (arrows indicate Golgi staining) and after 24 h (no staining in epithelium). Dotted lines indicate the cell surface. LP = lamina propria. Scale bars are 50 µm.

Mice were injected intraperitoneally with GalNAz and sacrificed every hour up to 12 h with an additional time point at 24 h. Intestinal sections were fixed in methacarn to preserve the mucus layer and stained for Muc2 (green) and GalNAz (red). The first indication of GalNAz incorporation was observed in the Golgi apparatus 1 h after injection, showing the initiation of *O*-glycosylation (Fig. 1, Supplementary Figs 1–3). In the crypts, maximum GalNAz staininig was observed after 4 h, demonstrating intense labeling of GCs and Paneth cells. The GalNAz signal continued to be present until 12 h and had mostly disappeared at 24 h. On the villi, the GalNAz signal was present early and the villi-resident GCs reached their maximum intensity at 3 h. None of the sections displayed any GalNAz signal in the villi epithelium at 24 h, indicating total turnover (Fig. 1, Supplementary Figs 1–3). Non-injected and vehicle-treated animals showed Muc2 staining similar to GalNAz-injected animals, indicating no adverse effects from GalNAz injection and labeling (Supplementary Fig. S12a,c).

### Muc2 turnover in goblet cells of villi is faster than in crypts

**Figure 2:**
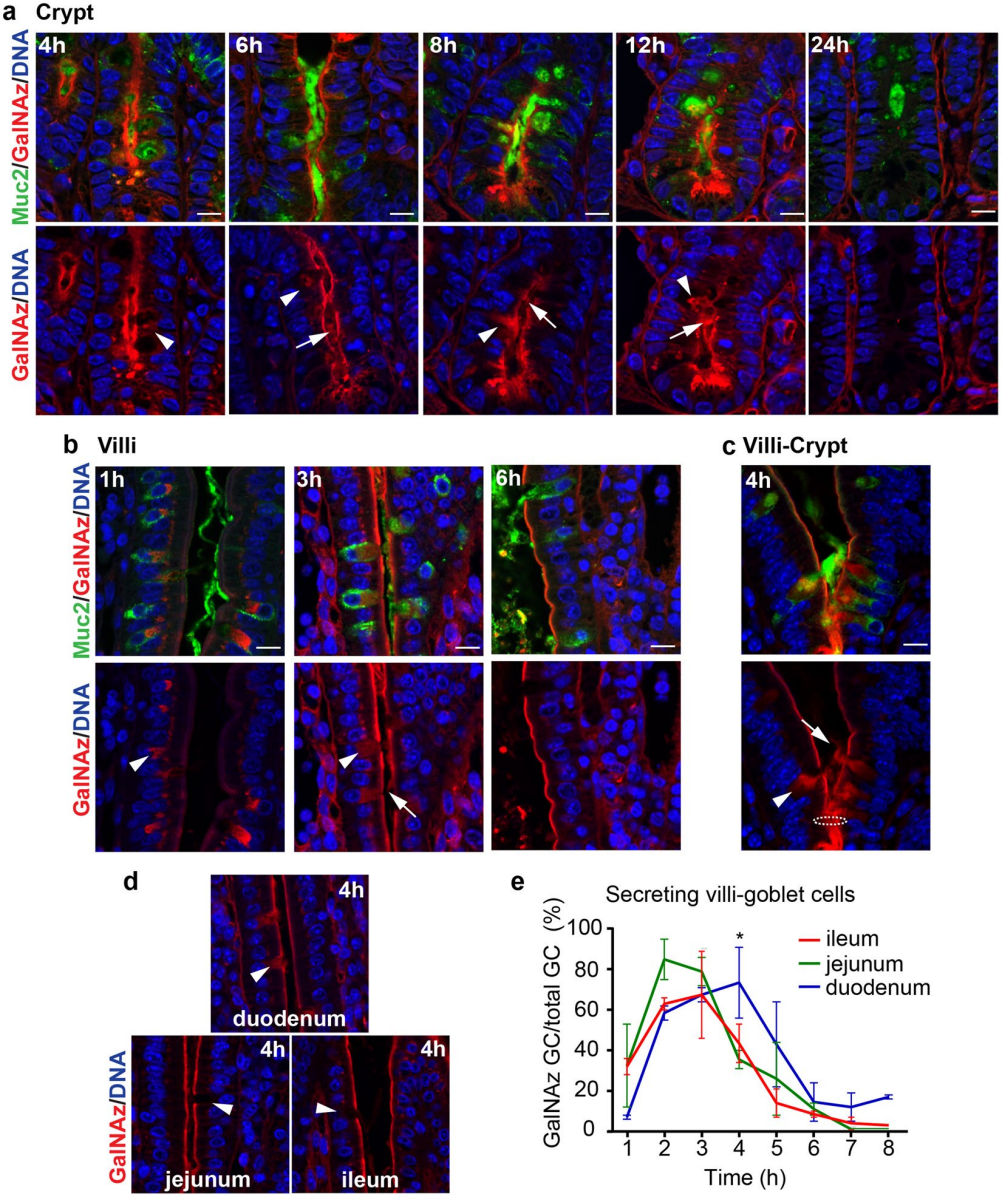
Ileal Muc2 secretion is faster at the villi than in the crypts. GalNAz-TAMRA (red) in comparison to immunostained Muc2 (green) in ileal sections. DNA detected with Hoechst in blue. **(a)** GalNAz-positive mucin granules in crypt GCs appear at 4 h after labeling. Stained Muc2 was secreted after 6 h and 12 h. **(b)** After 1 h, labeled Muc2 was observed in the Golgi of villi GCs. TAMRA-stained Muc2 was secreted at 3 h but was no longer observed after 6 h. **(c)** Fast producing and secreting GCs were observed just above the crypt opening (4 h). Intracellular labeled Muc2 is shown by arrowheads, secreted labeled mucus by arrows. Dotted circle indicates the crypt opening. Scale bars are 10 µm. **(d)** Muc2 secretion at 4 h at the different imntestinal locations. Arrowhead indicate GCs. **(e)** Quantification of GalNAz-Muc2 secreting GCs at the villi in relation to the total number of GCs. Most GCs were emptied after 2 h in ileum and jejunum with slower secretion in the duodenum. Significant differences were seen at 4 h. Data is presented as mean in % (*p > 0.05, n = 3 per time point).

In small intestinal crypts, GalNAz staining in the Golgi was observed as early as 1 h after injection (Fig. 1). However, GalNAz-positive mucin granules in GCs did not appear until after 4–5 h, indicating that mucin biosynthesis is a slow process which requires 3–4 h. Increased GalNAz signal in mucin granules together with secretion of stained mucus was seen at 8 h in ileum, duodenum and jejunum. Secreted mucus was still observed at 12 h in ileum but was completely absent at 24 h in ileum and jejunum with some residual staining detected in duodenum (Fig. 2a, Supplementary Figs S4a, S5a).

In all small intestinal sections, GCs on the upper villi surface also showed intracellular staining 1 h after injection (Fig. 2b, Supplementary Figs S4b, S5b). Stained structures included the Golgi apparatus but based on size, additional intracellular organelles such as *trans*-Golgi network and granules were likely also stained. Secretion of labeled mucus from GCs in villi was considerably faster than in the crypts and could be observed already at 3 h in all segments, but to a lesser extent in the duodenum (Supplementary Fig. S4b). After 4 h fewer secreting goblet cells in villi were present in jejunum and ileum compared to duodenum (Fig. 2d,e). After 6 h, secreted mucus was mainly observed in duodenum, and less so in jejunum and ileum (Fig. 2b, Supplementary Figs S4b, S5b). Quantification of secreting GCs confirmed slightly faster secretion in ileum and jejunum (2–3 h) as compared to duodenum (3–4 h) (Fig. 2e). The epithelium at and directly above the crypt opening contained several GCs that had a fast production and secretion of mucus, which was more similar to the GCs located on the upper villi than to GCs in the crypt. These cells secreted large amounts of stained mucus already 3–4 h after GalNAz injection (Fig. 2c, Supplementary Figs S4c, S5c).

Taken together, analysis of GalNAz-labeled Muc2 indicated that the production and secretion rate of small intestinal mucus was considerably faster in GCs on the villi compared to GCs in the crypt. This observation points to different functions for crypt and villi GCs in contributing to the small intestinal mucus protection.

### GalNAz labels Muc17 in the small intestinal glycocalyx

**Figure 3:**
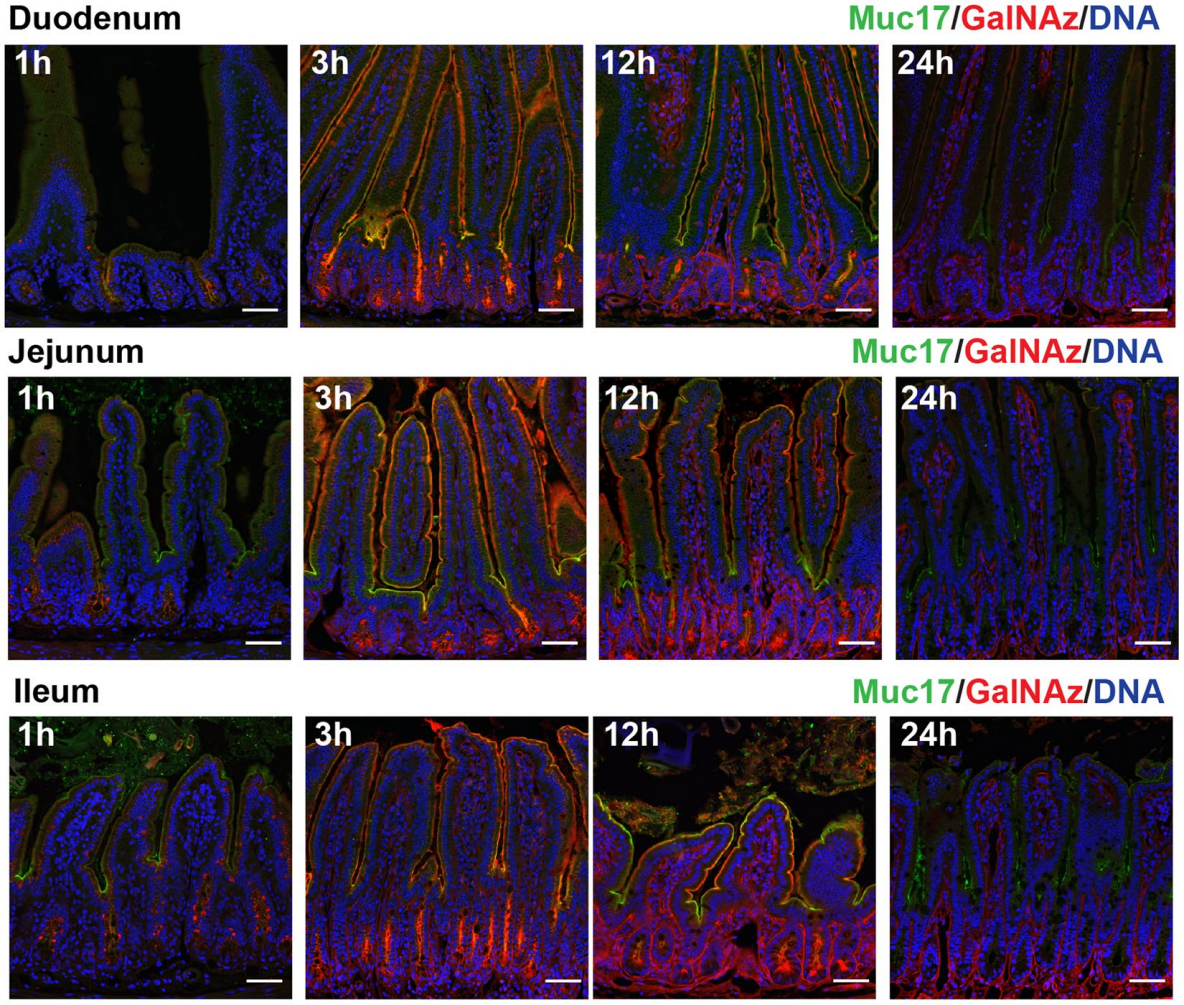
GalNAz-labeled Muc17 is expressed along the villi. GalNAz labeling of Muc17 was executed by intraperitoneal injection in mice, followed by TAMRA detection in methacarn fixed small intestinal sections. Muc17 was detected by immunostaining (green) along the villi and at the crypt openings, showing a characteristic patchy appearance. Hoechst DNA stain is shown in blue. GalNAz-labeled Muc17 was present at the villi at 3 h and 12 h throughout the small intestine, with no staining detectable after 24 h. Red signal in the crypts was already observed 1 h after GalNAz injection and intensifies at 3 h and 12 h (full sets are shown in Supplementary Figs 6–8). Scale bars are 50 µm.

The small intestinal glycocalyx covers the apical membrane of enterocytes, forming an additional dense barrier underneath the secreted mucus layer. Transmembrane mucins are extensively covered with glycan branches, each containing at least one GalNAc, and constitute an essential part of the glycocalyx. Although GalNAz in the small intestinal surfaces is incorporated in glycoproteins as well as glycolipids, the latter rarely contain GalNAc and thus contribute marginally to the observed GalNAz signal. In the mouse intestine, transmembrane mucin Muc17 is a major component of the glycocalyx and accounts for the bulk of incorporated GalNAz at the apical membranes. Immunostaining of Muc17 in duodenum, jejunum and ileum was performed in combination with GalNAz labeling, showing a typical patchy staining pattern restricted to apical surfaces of enterocytes (Fig. 3, Supplementary Figs 6–8. The apical surface of the epithelium was intensely stained by incorporated GalNAz, which was most prominent on villi tips and at crypt openings. The apical surface, both on villi and in crypts, showed strong GalNAz labeling already at 3 h and persisted for at least 12 h, but had completely disappeared at 24 h. This pattern was observed throughout the small intestine (Fig. 3, Supplementary Figs 6–8). Muc17 staining in non-injected and vehicle-injected animals showed no difference to GalNAz-injected animals, indicating that no adverse effects were introduced due to injection and labeling (Supplementary Fig. S12b, c).

### Highly glycosylated Muc17 is stably expressed at the plasma membrane

**Figure 4:**
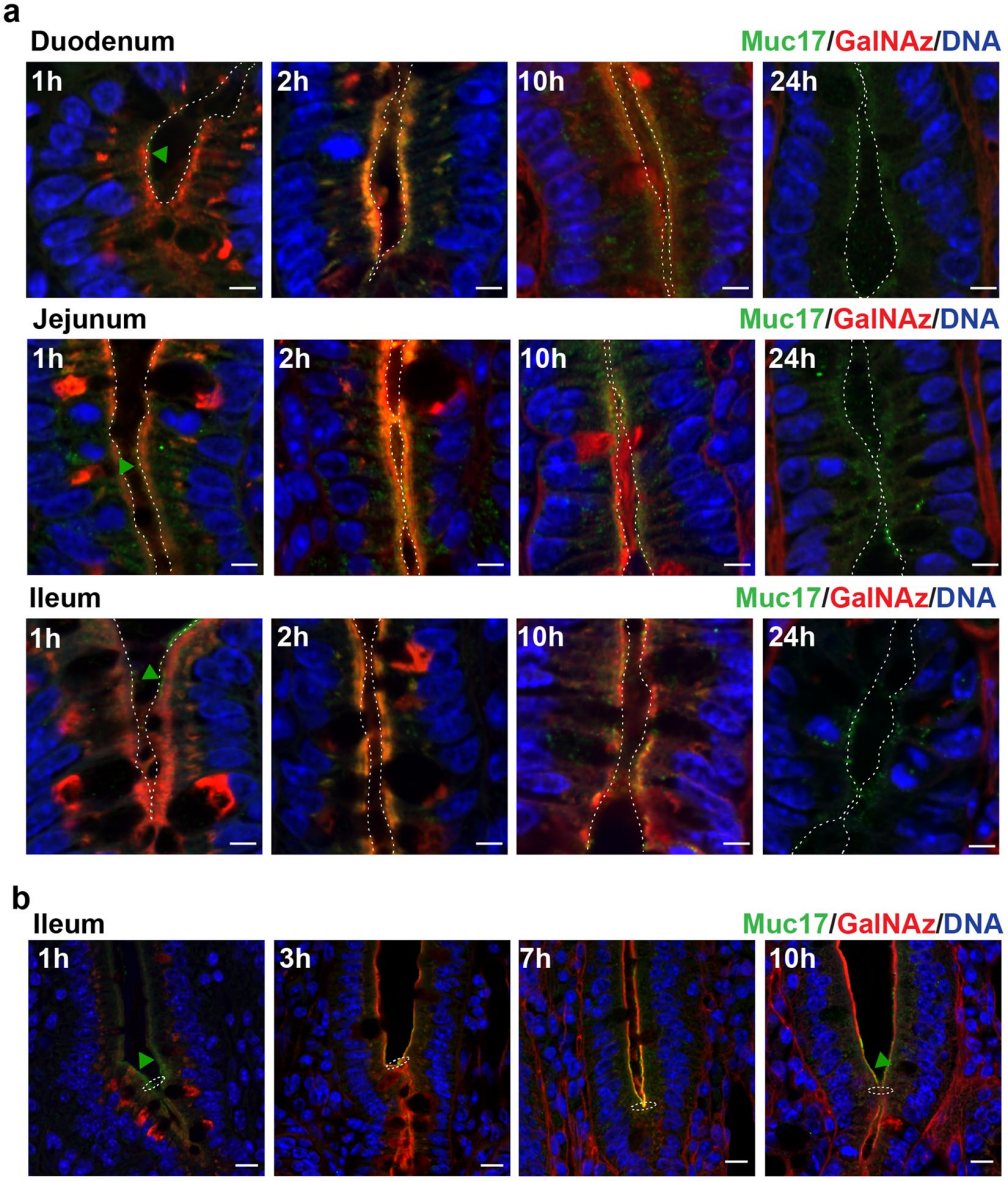
Muc17 is located at the apical surface of the crypt and above the crypt opening. (**a)** GalNAz-TAMRA (red), anti-Muc17 (green) and DNA (blue) staining of crypts in small intestinal sections. At 1 h, GalNAz staining was detected intracellularly and at the apical membrane. Green arrowheads point to immunostained Muc17 localized at the plasma membrane. 2 h post injection the GalNaz signal co-localized with the Muc17 immunostaining. A partial co-localization was still seen after 10 h, with no red staining after 24 h. Dotted lines indicate the cell surface. Scale bars are 5 µm. **(b)** In the ileum, GalNAz staining was present at 1 h on the apical epithelium above the crypt opening. Overlap of the GalNAz signal with Muc17 immunostaining at the apical plasma membrane can be detected at 3 h to 7 h. 10 h post injection GalNAz was still visible at the plasma membrane but no longer overlapped with the Muc17 immunostaining. The presence of immunostained Muc17 at the plasma membrane is pointed out by green arrowheads. Dotted circles indicate the crypt opening. Scale bars are 10 µm.

In the crypts, GalNAz staining was observed in the Golgi apparatus and subapically along the crypt surface as early as 1 h after injection (Fig. 4a). Notably, GalNAz labeling did not coincide with the apical Muc17 staining, indicating that GalNAz staining was representative of intracellular molecules. After 2 h the GalNAz and Muc17 staining overlapped, demonstrating insertion of GalNAz-labeled Muc17 into the apical plasma membrane. GalNAz-labeled Muc17 continued to be expressed at the plasma membrane up to 10 h after injection but had completely disappeared after 24 h (Fig. 4a). In the crypt-villi junction, Muc17 was clearly located at the apical surface where GalNAz staining appeared after 3 h and was still present after 10 h (Fig. 4b).

**Figure 5:**
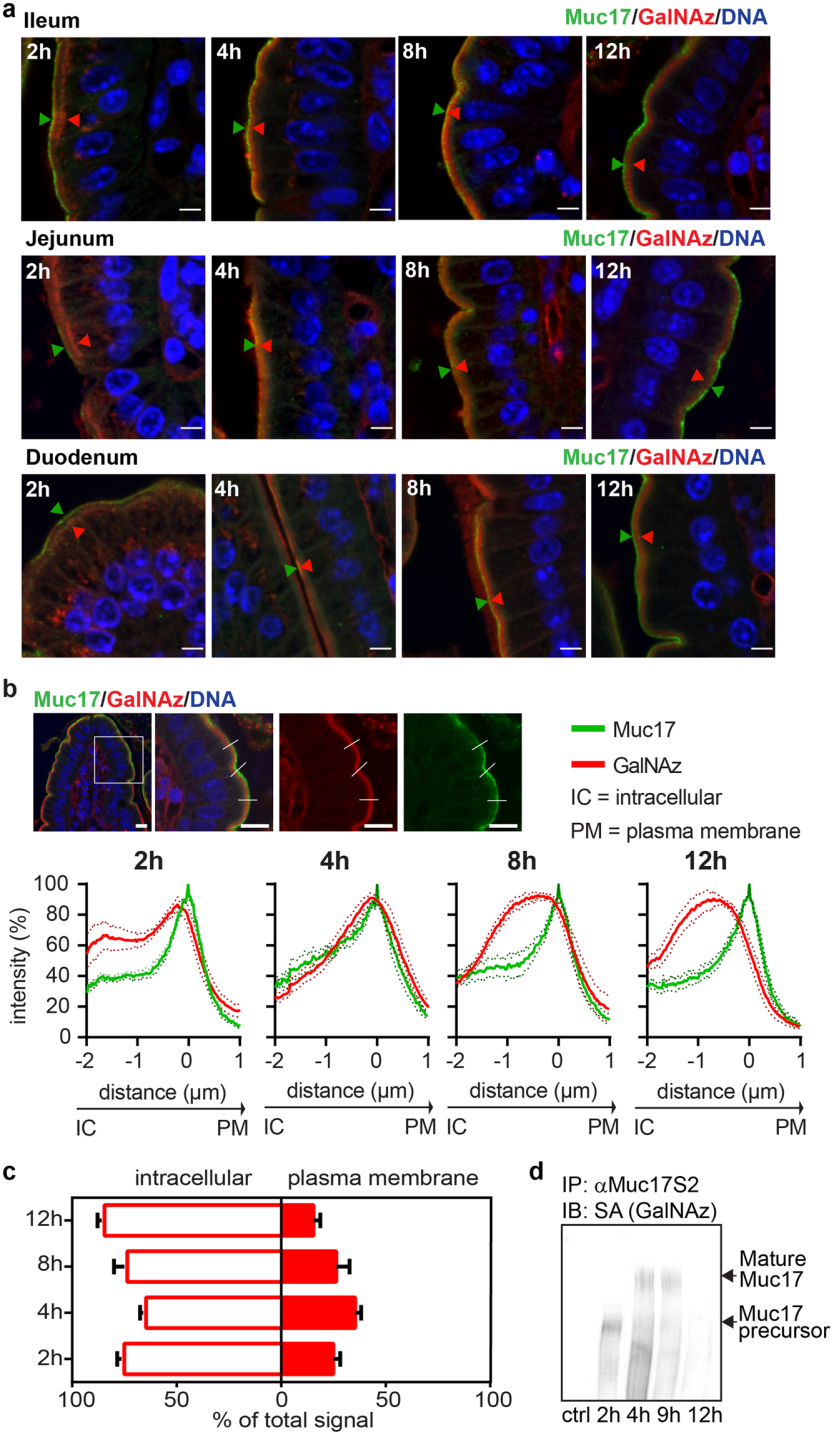
Muc17 is stably expressed apically in epithelial cells on the villi tip. (**a**) Close up view of upper villi epithelium with continuous Muc17 expression at the plasma membrane (anti-Muc17, green; DNA, blue). Intracellular GalNAz-labeled Muc17 (red) was seen at 2 h and overlapped with anti-Muc17 staining at the plasma membrane at 4 h and 8 h. After 12 h, GalNAz-Muc17 was again detected intracellularly where it localized close to the plasma membrane (a full set for ileum is shown in Supplementary Fig. S9). Arrowheads demonstrate the variation in localization of GalNAz-labeled Muc17 (red) in reference to immunostained Muc17 (green) at the plasma membrane. Scale bars are 5 µm. **(b)** Line profile quantification comparing the GalNAz signal with anti-Muc17 staining at villi tips. A representative image of an ileal section is shown, with the boxed in area displayed to the right and lines representing analyzed segments. Intensity profiles summarize quantification for all small intestinal regions with SEM (n = 5–6, individual line profiles are shown in Supplementary Fig. S10). Scale bars are 10 µm. **(c)** Representation of GalNAz-labeled intracellular and plasma membrane-localized Muc17 2 h to 12 h after GalNAz injection. **(d)** Muc17 was immunoprecipitated from small intestinal scrapings and analyzed by immunoblotting. GalNAz-biotin-labeled Muc17 was detected using streptavidin (SA), unlabeled samples served as negative control. A large molecular weight band of mature Muc17 was detected at 4 h and 9 h, with a smaller band visible at 2 h, likely representing a less glycosylated precursor.

On the upper villi, GalNAz-labeled Muc17 was observed intracellularly, most likely in the Golgi apparatus (Fig. 5a, Supplementary Fig. S9). At 2 h, GalNAz stained the subapical region, followed by staining at the plasma membrane 4 h and 8 h after injection where it appeared together with immunostained Muc17. GalNAz was absent from the Muc17-positive apical surface at 12 h and no apical GalNAz signal was observed after 24 h, indicating complete turnover. Line profile quantification of representative images from sections along the upper villi confirmed the described microscopic observations, showing maximum overlap between GalNAz and Muc17 signals in the apical plasma membranes 4 h post injection (Fig. 5b,c, Supplementary Fig. S10). The specificity of GalNAz incorporation into Muc17 was verified by detecting GalNAz in immunoprecipitated Muc17 from scraped mouse intestines using a Click-iT^TM^ biotin reagent. Biotinylated GalNAz was present as a high molecular weight band in tissues collected at 4 h and 9 h post injection (Fig. 5d, Supplementary Fig. S11). GalNAz was also detected in a fast migrating, not fully glycosylated band observed after 2 h. At 12 h, a weak GalNAz-positive band remained, indicating decreased GalNAz-labeled Muc17 in line with decreased overlap of Muc17 and GalNAz staining in tissue sections at this time point (Fig. 5b). The high molecular weight Muc17 remained labeled and stable for at least 5 h, indicating fast renewal (Fig. 5d, Supplementary Fig. S11).

## Discussion

Throughout the intestine, adult stem cells reside far down in the crypts, rendering the crypts the most vulnerable sites of the epithelium. In the small intestine, Paneth cells secrete antibacterial molecules into the lumen in order to ensure protection of neighboring crypt stem cells. More differentiated GCs in the upper crypt and along the villi secrete mucus to form a diffusion barrier, thus protecting the intervillar region. The loose small intestinal mucus can readily trap the luminal content and transport it distally in coordination with motility. By contrast, in the colon which lacks Paneth cells, sentinel GCs guard the crypt openings^19^.

Previous electron microscopy studies have identified GCs as common or granular mucous cells, with the latter residing mainly in the upper crypt^8^. In contrast to these findings, our study suggests the existence of several distinct types of GCs in the small intestine, characterized by differences in their biosynthesis machinery as well as secretion pattern and timing. Similar observations were previously reported in the colon, where GCs at the luminal surface secreted mucus much faster than their counterparts along the crypts^18^. Based on mucus-producing properties, we have chosen to distinguish between two main subset of GCs, termed crypt-type GCs and villi-type GCs. The upper crypt features GCs with large, mucus-filled theca corresponding to granular cells. Biosynthesis and granule storage in these cells was slow as GalNAz-labeled intracellular material was not visible until after 4–5 h post injection. Secretion of GalNAz-labeled mucus was first observed after 6 h and continued over 6–8 h with labeled granules still present after 12 h. Some stained vesicles even remained after 24 h in the duodenum. Based on the observation that there was no universal GalNAz incorporation in all granules at any specific time point, it seems likely that different granules resides in the theca for various times, resulting in a combination of GalNAz-positive and -negative vesicles together with secretion of GalNAz-labeled material. Therefore, we classify GCs located at the upper crypt as crypt-type GCs, a distinct subpopulation in which mucus is stored to be massively secreted if the crypt is in danger. This is in analogy with sentinel GCs of the colon^19^. It is noteworthy that the crypt-type GCs in the upper crypt are totally absent from the villi epithelium, indicating that villi-type GCs empty their mucus content in bulk. In summary, the critical crypt openings are safeguarded by dense mucus from crypt-type GCs and hold high concentrations of antibacterial molecules produced by Paneth cells that trap, neutralize and repel invading bacteria.

The crypt-villus interface lacks mucus-filled GCs, which are instead replaced by a subpopulation of GCs with higher production and secretion rate. Mucus secretion is first observed at this location around 3 h after GalNAz injection, indicating a faster turnover than in crypt-type GCs. The existence of these cells could either be explained by emptying of mucus content followed by shedding or by conversion of crypt-type CGs to villi-type GCs. A transformation process is most likely as we do not observe any accumulation of dead cells in this region, in accordance with results from previous studies^8^. Instead, cells expelled from the epithelium are observed at the villi tip where epithelial cells undergo anoikis and are ejected, normally without impairing the epithelial integrity^20^. If a transition between these crypt-type and villi-type GCs takes place, reprogramming is likely tightly regulated and confined to a precise region along the crypt-villus axis.

GCs located at the upper villi produce mucus at a similar rapid rate as observed for lower villi-type GCs. Initial mucus secretion is observed after 2 h, with few GalNAz-labeled cells remaining 5 h after GalNAz injection. The prompt production and secretion of mucus could be implicated in the fast motility of luminal material^21^; within 1 h molecules residing in the Golgi progressed to secretion. Fast mucus renewal in GCs was previously observed after stimulation of compound exocytosis, followed by replenishing the mucin pool within 1 h^22^. In our study, mucus renewal in villi-type GCs occurred within 4 h. If cell migration along the villi takes about one day, each GC could empty and refill its mucus content 6 times within that period. Glycan-specific immuno-electron microscopy data suggest mucus renewal for each cell to occur approximately 4 times, but this process will be influenced by the glycosylation within cells^23^.

Although our data point to an extremely fast rate of mucus renewal in the inter-villi region, a potential stimulatory influence of the intraperitoneal injection cannot be dismissed. Older data obtained from rabbits indicate that a core of granules remains in the center of the villi GCs^23^, but most of the theca were observed with GalNAz-labeled mucus at some point. This suggests a constant and fast renewal of the mucus secreted from villi located cells. A slight difference in turnover was estimated along the small intestine with a slower process in duodenum where most mucus-secreting GCs on the villi epithelium were observed after 4 h and continued to be present for at least two additional hours.

GalNAz readily labels *O*-glycosylated structures, including GCs as well as transmembrane mucins. The apical surface epithelium was intensely stained after 2 h and remained labeled up to 12 h post injection. The staining was abolished after 24 h revealing a very stable expression of glycosylated molecules with a turnover of less than a day. Immunostaining demonstrated Muc17 to be predominantly expressed at the upper villi region, around the crypt opening and in the crypt. In the crypts, Muc17 is either observed on the apical surface or in intracellular vesicles, possibly due to recycling^13^. We initially detected immunostained apical Muc17 luminal to the GalNAz signal. After 2 h, Muc17 and GalNAz staining overlapped and continued to coincide until 12 h, again indicating stable plasma membrane expression of labeled material.

At the villi surface above the crypt openings, overlapping staining of Muc17 and GalNAz was present 3–7 h after labeling and decreased after 12 h, pointing to a faster turnover rate. On villi tips, GalNAz was observed subapically before 2 h, after which stainings converged to varying degrees. Muc17 staining and GalNAz labeling diverged at 12 h, indicating that Muc17 turnover in the villi is faster in comparison to other glycosylated apical membrane components. This was further confirmed by immunoblotting of GalNAz-labeled Muc17 from small intestinal scrapings, showing almost no labeled MUC17 at 12 h.

**Figure 6:**
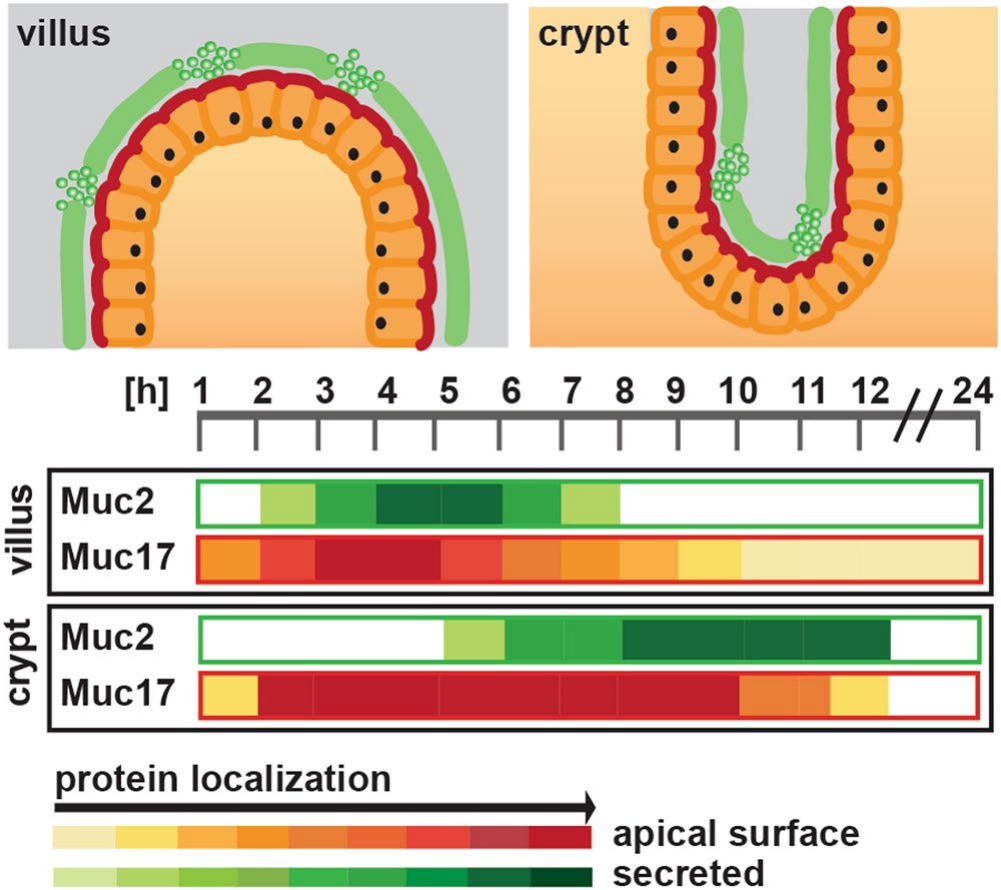
Overview of Muc2 and Muc17 turnover in the small intestine. Schematic representation of Muc2 secretion and Muc17 plasma membrane localization in small intestinal crypts and villi. Both processes occur rapidly at the villi surface, but the Muc2 secretion was more transient than the Muc17 surface localization. In the crypt, Muc2 secretion occurs later and slightly slower, whereas Muc17 plasma membrane localization was equally fast, but extends over a much longer time.

In conclusion, we have used an *in vivo* labeling approach to study the turnover of secreted Muc2 and plasma membrane-tethered Muc17 throughout the mouse small intestine. We have demonstrated a slower mucus production in mucus-accumulating GCs in the crypts compared to GCs located along the villi. Villi-type GCs rapidly produce and secrete mucus, frequently replenish the inter-villar mucus and thus protect the intestinal epithelium. Transmembrane mucin Muc17, forming the glycocalyx, was mainly expressed at the villi tip, above the crypt opening and in the crypt where it was stably localized at the plasma membrane as a highly glycosylated product for at least 5 h (Fig. 6).

## Methods

### Animals and ethics statement

All mice in this study were inbred on the C57Bl/6 background and kept under standardized conditions of temperature (21–22 °C) and illumination (12 h light/12 h dark). Food and water was provided ad libitum. Animal experimental procedures were approved by the Swedish Laboratory Animal Ethical Committee in Gothenburg (reference number 358–2009) and were conducted in accordance with guidelines of the Swedish National Board for Laboratory Animals.

### Intraperitoneal injection of GalNAz in mice and tissue fixation

GalNAz (Invitrogen) was injected intraperitoneally in mice as described previously^18^. In short, 2.6 mg GalNAz dissolved in 25 µl DMSO was diluted in PBS to a final volume of 500 µl for each animal. Injections were given intraperitoneally within the dark period (04:00 a.m.). Three animals were sacrificed every hour up to 12 h after injection with an additional 24 h time point. The intestine was removed and pieces of the first (duodenum), middle (jejunum) and last (ileum) portion of the small intestine containing luminal material were fixed in methacarn (methanol Carnoy) (60% (v/v) dry methanol, 30% (v/v) chloroform, 10% (v/v) glacial acetic acid). Paraffin embedding was preceded with a methanol wash and 4 μm sections were cut and placed on glass slides.

### Fluorescent detection of GalNAz in fixed tissue and mucin staining

Detection of the azide labeled *O*-glycans was performed by conjugating the azide to a fluorescently labeled alkyne in a copper-catalyzed azide-alkyne cycloaddition reaction^24^ as previously described^18^. Fixed paraffin embedded sections were dewaxed, hydrated, and washed in PBS. Tissue sections were incubated with 10 µl of reaction mix from the tetramethylrhodamine (TAMRA) glycoprotein detection kit (Invitrogen) and incubated at 4 °C overnight. After washing in PBS, the samples were blocked in 5% (v/v) fetal bovine serum and stained with anti-MUC2C3 antiserum (against a C-terminal peptide)^25^ or anti-Muc17S2 (against a peptide in the SEA domain)^26^. The specific antibodies were detected with an Alexa 488 conjugated anti-rabbit antibody (Invitrogen) and the sections were mounted using ProLong anti-fade mounting medium (Invitrogen). The images were acquired using a LSM 700 Axio Examiner.Z1 laser scanning confocal microscope, with Plan-Apochromat 20x/0.8 and 40x/1.3 Oil DIC objectives (Zeiss). The images were acquired and processed uniformly using the ZEN 2012 software (Zeiss).

### Immunoprecipitation and biotin labeling of Muc17

The small intestinal epithelium was scraped from 5 cm segments taken between the ileum and jejunum from animals 2, 4, 9 and 12 h after GalNAz-injection. The scrapings were frozen in small volumes of PBS with 2x cOmplete^TM^ Protease Inhibitor Cocktail (Roche). Immunoprecipitation of Muc17 was performed on ice. Magnetic Protein G Dynabeads (Life Technologies) were washed in PBS and coated with anti-Muc17S2 antibody in PBS at 4 °C overnight. Unbound antibodies were removed by washing in PBS and beads equilibrated in PBS with 1% (v/v) Igepal. Tissue samples were re-suspended in high salt lysis buffer (50 mM Tris pH 7.5, 1 M NaCl, 10 mM MgCl_2_, cOmplete^TM^ Protease Inhibitor Cocktail, 5% (v/v) glycerol, 1% (v/v) Triton-X 100, 1 mM EDTA), mechanically lysed and debris removed by centrifugation at 2,000 g and 4 °C for 10 min. Supernatants were bound to Protein G Dynabeads at 4 °C overnight. Unbound material was removed by washing in PBS with 1% (v/v) Igepal.

Click-iT^TM^ reactions were performed on immunoprecipitation beads with anti-Muc17S2 bound products using the Click-iT^TM^ Biotin Protein Analysis Detection Kit (Life Technologies) according to the manufacturer’s instructions. Briefly, to each sample 5 µl Biotin alkyne with 100 µl Click-iT^TM^ reaction buffer and 60 µl H_2_O were added and vortexed. 10 µl CuSO_4_ were applied, vortexed, then 10 µl Click-iT^TM^ reaction buffer additive 1, vortexed and incubated for 3 min at room temperature (RT). 20 µl Click-iT^TM^ reaction buffer additive 2 were added and incubated at 4 °C overnight. Samples were washed in PBS with 1% (v/v) Igepal and eluted in 2x loading buffer (100 mM Tris-HCl pH 6.8, 4% (w/v) SDS, 20% (w/v) glycerol, 200 mM DTT, 0.2% (w/v) bromophenol blue) at 95 °C and centrifuged at 1,400 rpm for 5 min.

### Electrophoresis and western blot

Samples were separated on 5% or 10% polyacrylamide gels and transferred to PVDF membranes (Millipore) using semi-dry blotting procedures. For detection of Muc17, membranes were blocked in 5% (w/v) solution of powdered milk and incubated with primary antibodies, anti-Muc17C1 (1/1,000) or anti-Muc17S1 (1/1,000)^26^ at 4 °C overnight. Secondary antibody incubation was conducted for 2 h at RT with goat-anti-rabbit HRP (1/10,000, Southern Biotech) and the membranes were developed using a chemiluminescent HRP substrate, Immobilon (Millipore). For detection of GalNAz labeled products, membranes were blocked with 3% (w/v) BSA solution, incubated with alkaline phosphatase conjugated streptavidin (1/5,000, Southern Biotech) for 2 h at RT and blots developed with NBT-BCIP color reagent (Promega).

### Data presentation and statistical analysis

Mucus-secreting GCs were counted on an area of 10 villi from two to three animals per time point. Total numbers of secreting GCs were determined by anti-MUC2C3 staining (green). Quantification of cells secreting *de novo* synthesized mucus was based on red TAMRA-GalNAz stain in the same villi. The ratio of villi-resident, GalNAz-secreting GCs (red) to total number of secreting GCs (green) was calculated. Data is presented as mean % and statistical analysis performed on the arcsine transformed ratio with two-way ANOVA and Holm-Šídák test for multiple comparisons.

Intensity profiles of GalNAz-TAMRA and Muc17 in intestinal sections from villi tips were generated using ImageJ 1.49 v. Data from three line profiles were collected from each image. Line intensities were normalized, averaged and plotted against distance along the intracellular-extracellular axis crossing the apical plasma membrane. Muc17 in the plasma membrane was defined as showing maximum intensity and set to 0 µm along the intracellular-extracellular axis. The ratio of GalNAz-labeled Muc17 (red) to total Muc17 (green) was determined by calculating the area under the curve for intracellular and plasma membrane-resident GalNAz-labeled and total Muc17, respectively. Data is presented as mean ± SEM.

### Data availability

All data generated or analyzed during this study are included in this published article (and its Supplementary Information files).

## Electronic supplementary material

Supplementary figures S1-S12
