# Supplementary material for: Study of mucin turnover in the small intestine by in vivo labeling
Source: Sci Rep. 2018 Apr 10;8:5760. doi: 10.1038/s41598-018-24148-x (PMC5893601; doi:10.1038/s41598-018-24148-x)
Supplement: Supplementary file 1 — Supplementary figures S1-S12 [file 41598_2018_24148_MOESM1_ESM.pdf]

Study of mucin turnover in the small intestine by *in vivo* labeling

Hannah Schneider, Thaher Pelaseyed, Frida Svensson and Malin E.V. Johansson

Department of Medical Biochemistry and Cell Biology, University of Gothenburg, Sweden

SUPPLEMENTARY FIGURE LEGENDS

Supplementary Fig. S1. GalNAz-labeled Muc2 in the duodenum is expressed after 1 h in the crypts and along the villi. Incorporated GalNAz detected by TAMRA (red) and immunostaining of Muc2 (green) with Hoechst DNA stain (blue) on duodenal sections 1-24 h after intraperitoneal injection. Images for crypts and villi are displayed in separate panels. In duodenal crypts, GalNAz-labeled Muc2 was detected in the Golgi apparatus 1 h after injection, with onset of secretion at 2 h. Labeled Muc2 continues to be present until 12 h post injection but was absent at 24 h. At the villi, intracellular GalNAz was expressed at 1 h after labeling. First secretion of labeled Muc2 was seen at 3 h and increased in intensity over time. No GalNAz signal was present after 24 h.

Supplementary Fig. S2. Muc2 production and secretion happens rapidly in the jejunum. Jejunal sections stained for Muc2 (green) and DNA (blue). Incorporated GalNAz was detected using TAMRA (red). After 1 h, some intracellular GalNAz signal was seen in cells in the crypts and along the villi. The staining intensified at 2 h post injection and secretion of labeled Muc2 could clearly be observed after 3 h. GalNAz staining was continuously detected until 12 h after injection but was mostly absent after 24 h.

Supplementary Fig. S3. Muc2 secretion in ileum coincides with observations from duodenum and jejunum. Incorporated GalNAz detected by TAMRA (red) and immunostaining of Muc2 (green) with Hoechst DNA stain (blue) on ileal sections 1-24 h after injection of GalNAz. Villi and crypts are positive for GalNAz labeling 1-12 h after labeling. Initial staining was observed in the Golgi at 1-2 h, followed by secretion of labeled material at later time points. Most intense staining was seen at 4 h after injection and no GalNAz signal was present after 24 h.

Supplementary Fig. S4. Mucus biosynthesis in duodenal crypts and villi occurs at different rates. GalNAz-TAMRA (red), anti-Muc2 (green) and DNA (blue) staining in duodenal sections. (a) GalNAz-TAMRA stained mucin granules 4-6 h after GalNAz injection in the crypt GCs, followed by secretion of stained mucus at 8 h which continued until 12 h. (b) GCs along the villi contained intracellular GalNAz labeling after 1 h, with secretion of labeled mucus after 3 h continuing up to 6 h. (c) GCs in the villus-crypt junction secrete GalNAz-labeled mucus comparable to villi GCs (after 3 h). Arrowheads point to intracellular GalNAz-stained Muc2, whereas arrows indicate secretion of labeled mucus. Dotted circle indicates the crypt opening. Scale bars are 10 µm.

Supplementary Fig. S5. Muc2 turnover in villus and crypt located GCs in the jejunum. GalNAz-TAMRA (red), anti-Muc2 (green) and DNA (blue) staining of crypt and villus sections in jejunum. (a) Crypt GCs were positive for GalNAz in some mucin vesicles 4 h after injection.

Secretion of labeled Muc2 was first observed after 6 h and continued until 12 h. (b) In the villus GCs, intracellular GalNAz staining was seen in the Golgi already 1 h after injection. Labeled Muc2 was first secreted after 3 h and was mostly absent after 6 h. (c) Fast secreting GCs were observed in the villus-crypt region (3 h). Intracellular GalNAz-labeled Muc2 is shown by arrowheads and secreted GalNAz labeled Muc2 is pointed out by arrows. Dotted circle indicates the crypt opening. Scale bars are 10 µm.

Supplementary Fig. S6. Duodenal villi are intensely stained for GalNAz-labeled Muc17. In duodenal sections incorporated GalNAz was detected using TAMRA (red), together with immunostaining of Muc17 (green) and DNA (blue). Sections for villi and crypts are displayed in separate panels. Expression of GalNAz labeled Muc17 was observed between 2-12 h and was most intense at the villi tips and above the crypt openings. At 2-3 h the GalNAz staining overlapped with the Muc17 immunostaining, indicating plasma membrane localization. The GalNAz signal intensified at later time points up to 12 h but was absent at 24 h.

Supplementary Fig. S7. Muc17 is expressed over several hours along the jejunal villi. Overview of GalNAz detected by TAMRA (red) together with immunostaining of Muc17 (green) and DNA (blue) in sections from jejunum 1-12 h and 24 h after intraperitoneal injection of GalNAz. Muc17 was expressed in a characteristic patchy pattern along the villi and above the crypt openings. Intracellular GalNAz was seen at 1-2 h followed by detection at the plasma membrane at 3 h post injection. The surface labeling continued to be present until 12 h and was most prominent along the villi. No GalNAz staining remained after 24 h.

Supplementary Fig. S8. GalNAz-labeled Muc17 is localized at the ileal plasma membrane 4 h after injection. Incorporated GalNAz detected by TAMRA (red) and immunostaining of Muc17 (green) with Hoechst DNA stain (blue) on ileal sections 1-24 h after intraperitoneal injection of GalNAz. In the crypts and in few cells along the villi intracellular GalNAz staining was present at 1 h after injection. Beginning from 3 h, GalNAz and immunostaining of Muc17 overlapped at the plasma membrane, which was most obvious after 4 h. Plasma membrane localization of labeled Muc17 continued to be present over the following time, with partial internalization of the signal until 12 h. No staining was detected at 24 h after labeling.

Supplementary Fig. S9. Muc17 is expressed at the plasma membrane along the villi tips in the ileum. Incorporated GalNAz detected by TAMRA (red) and immunostaining of Muc17 (green) with Hoechst staining of DNA (blue). Shown are representative images of the upper villi epithelium in ileum with zoomed in sections displayed in the lower panel. 1 h after injection GalNAz was detected in the Golgi, followed by expression close to the plasma membrane after 2 h. At 4 h, Muc17 staining at the plasma membrane overlapped with GalNAz labeled Muc17 and continued to do so until 10 h. GalNAz was seen in the terminal web region at 12 h and was absent at 24 h.

Supplementary Fig. S10. Line profile quantification of Muc17 localization at the plasma membrane of villi tips. Intensity profiles of Muc17 (green) and GalNAz (red) labeling at the villi tips in duodenum, jejunum and ileum 2 h, 4 h, 8 h and 12 h after GalNAz injection. Representative profiles for each time point and localization are shown. Overlapping curves indicate plasma membrane localization of GalNAz-labeled Muc17, which was most prominent at 4 h. At earlier and later time points a high proportion of GalNAz labeled Muc17 was localized intracellularly.

Supplementary Fig. S11. Muc17 was identified in intestinal scrapings following enrichment by immunoprecipitation. Small intestinal scrapings were used for immunoprecipitation of Muc17 and subsequent detection by immunoblotting. The input of the lysate was too low to detect Muc17 (left), but after immunoprecipitation two bands were seen using Muc17S1 antiserum (arrows, right), indicating products with different glycosylation pattern.

Supplementary Fig. S12. Control staining of Muc2 and Muc17 in non-injected and vehicle injected mice. Mucin staining (green) with anti-MUC2C3 antibody (a) and anti-Muc17S2 antibody (b) in the three small intestinal segments of non-injected control mice. Hoechst was used to stain DNA (blue). (c) Muc2 and Muc17 staining of vehicle injected mouse ileal samples.Arrows indicate Muc2 secretion from surface GC and arrowheads indicate Muc2 filled upper crypt GCs. Stars indicate intense Muc17 staining at the crypt-villus junction.

Figure S1

Duodenum

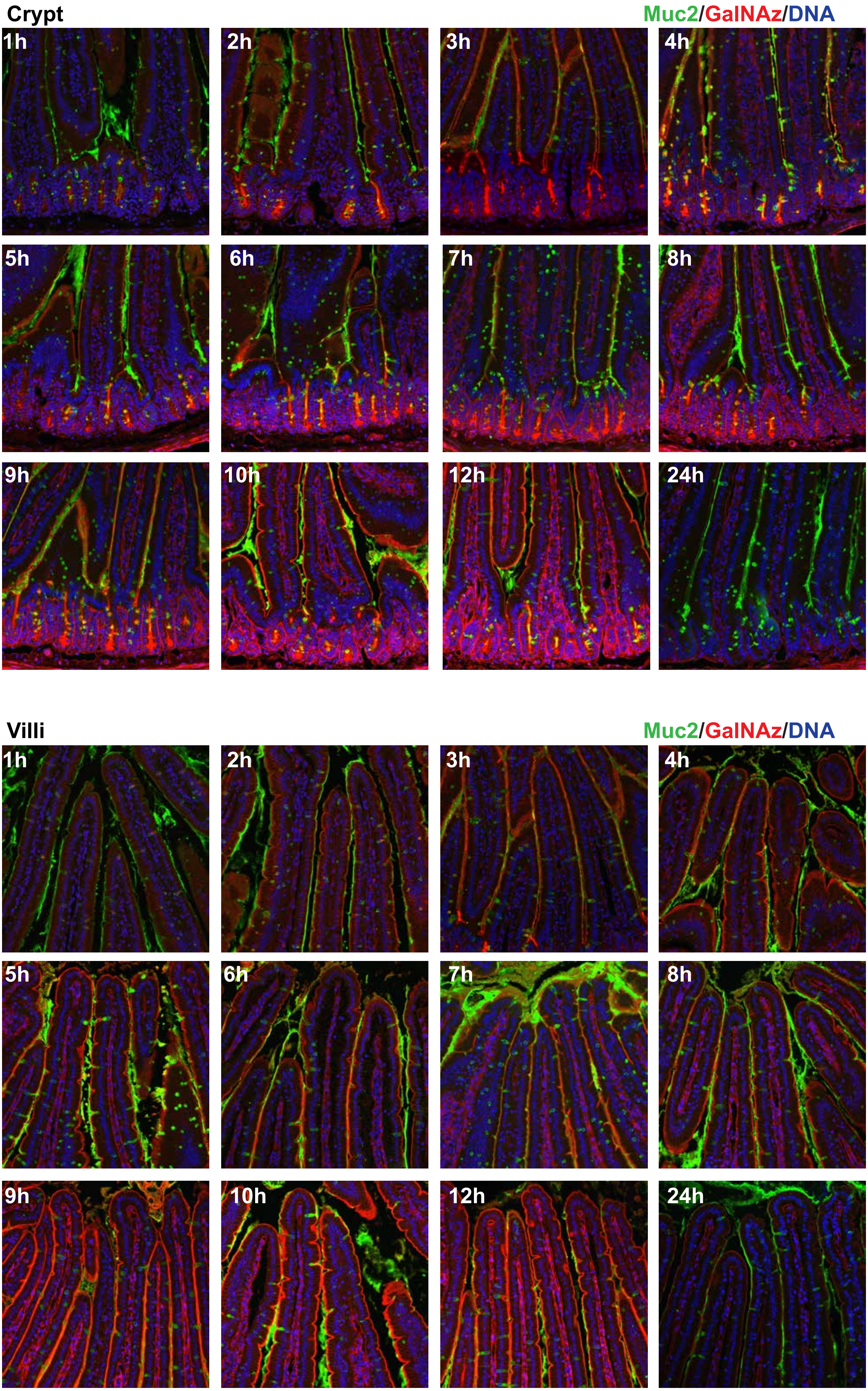

Figure S2

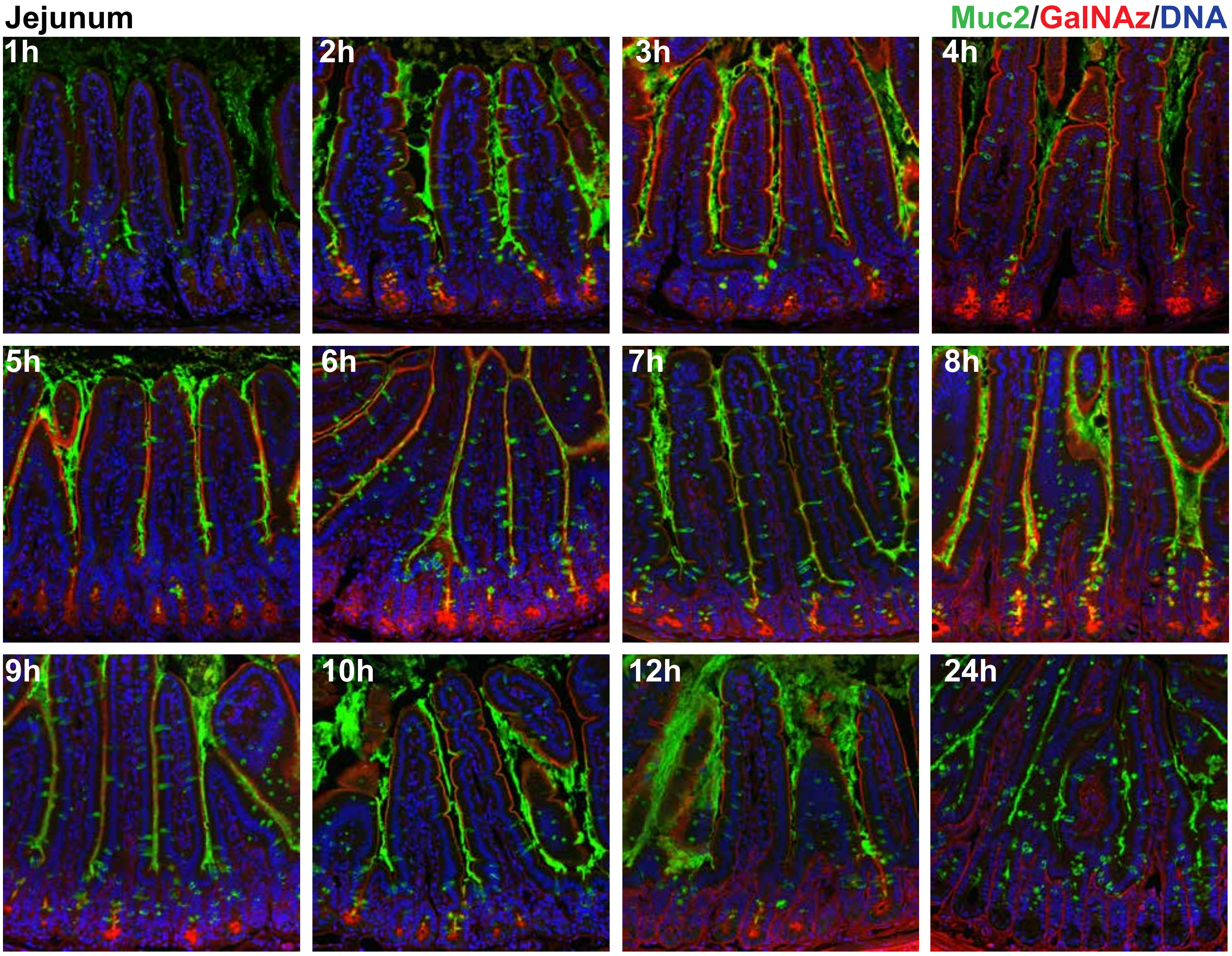

Figure S3

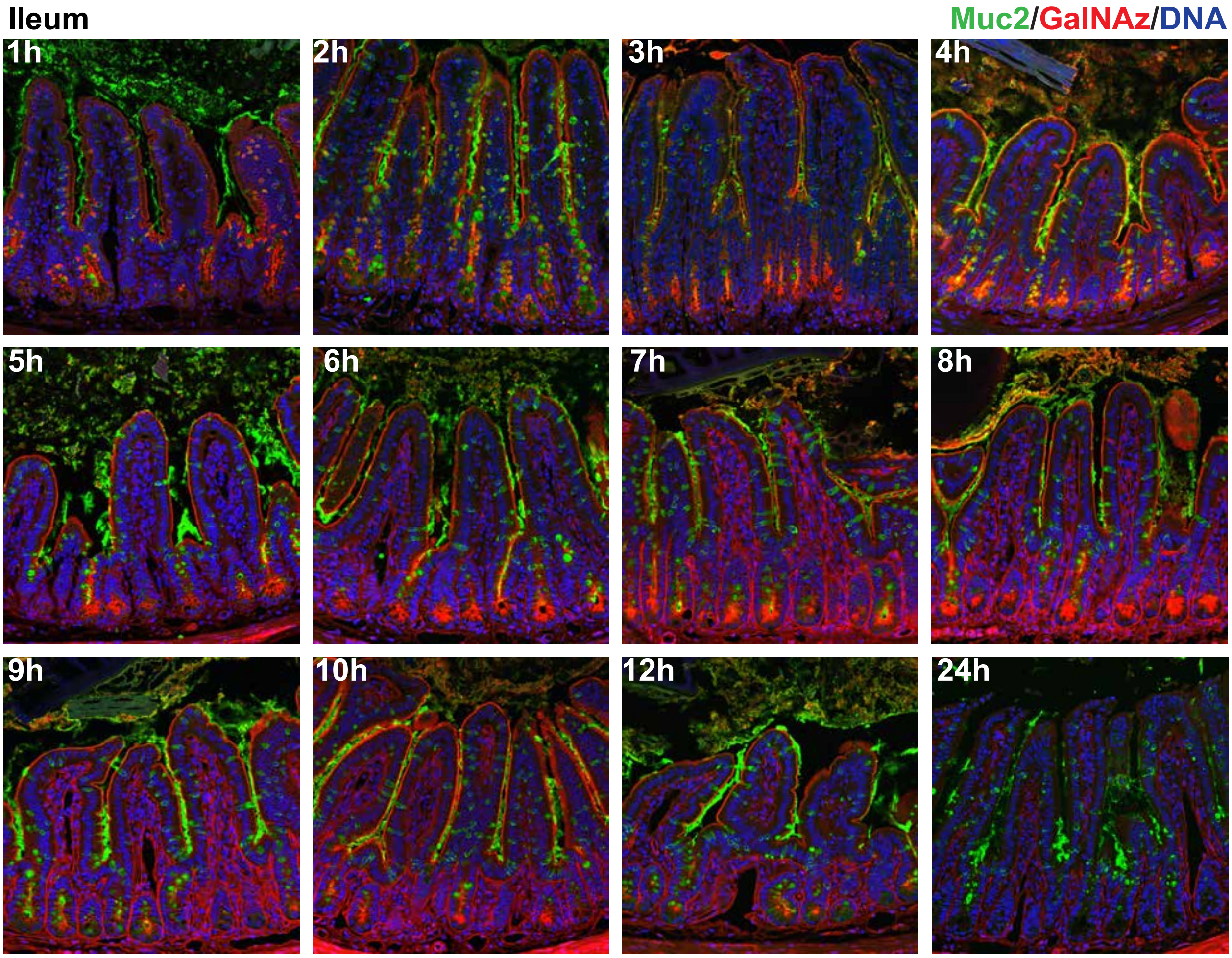

Figure S4

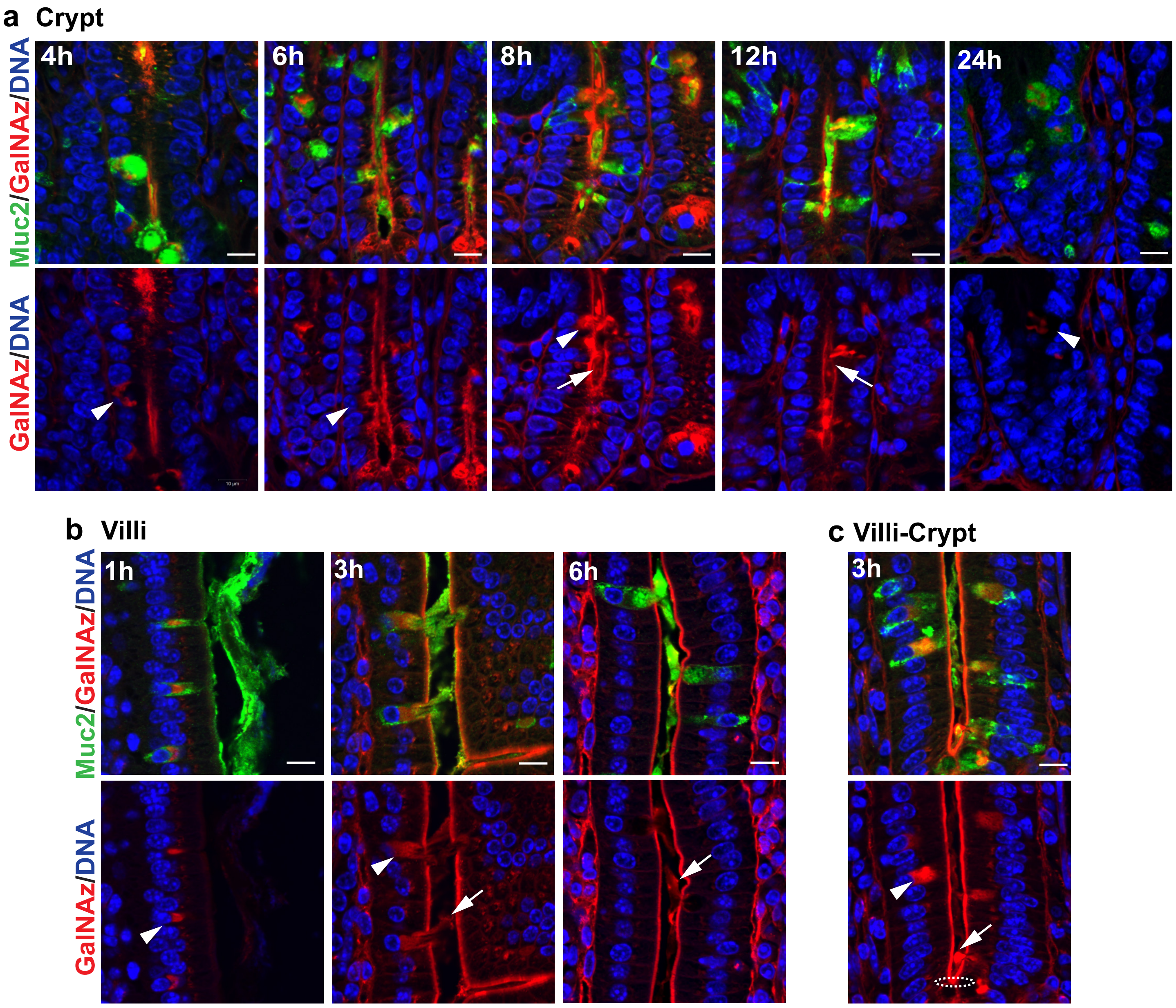
a Crypt
Muc2/GalNAz/DNA
GalNAz/DNA
4h
6h
8h
12h
24h
b Villi
1h
3h
6h
c Villi-Crypt
3h

Figure S5

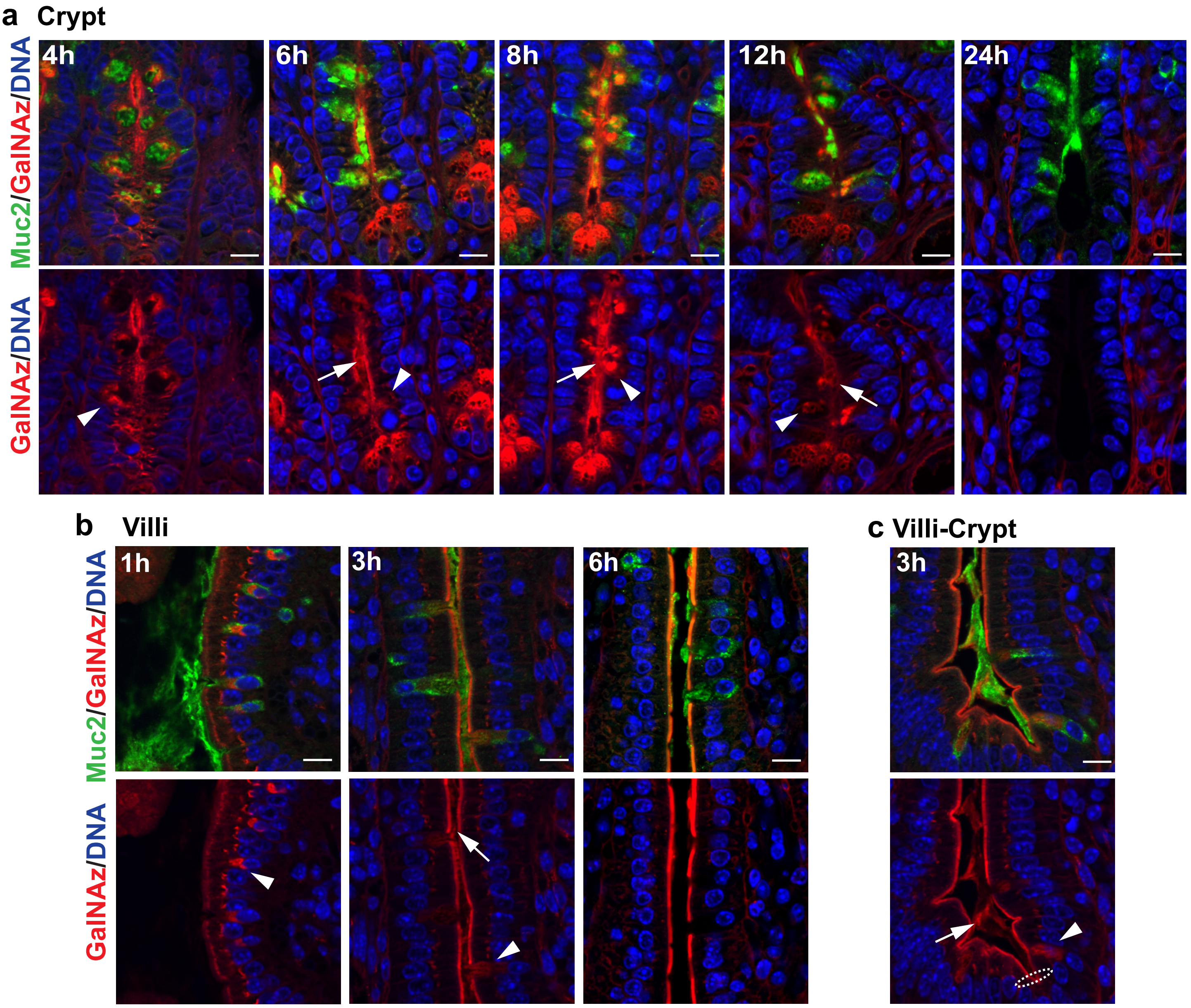
a Crypt
Muc2/GalNAz/DNA
GalNAz/DNA
4h
6h
8h
12h
24h
b Villi
Muc2/GalNAz/DNA
GalNAz/DNA
1h
3h
6h
c Villi-Crypt
3h

Figure S6

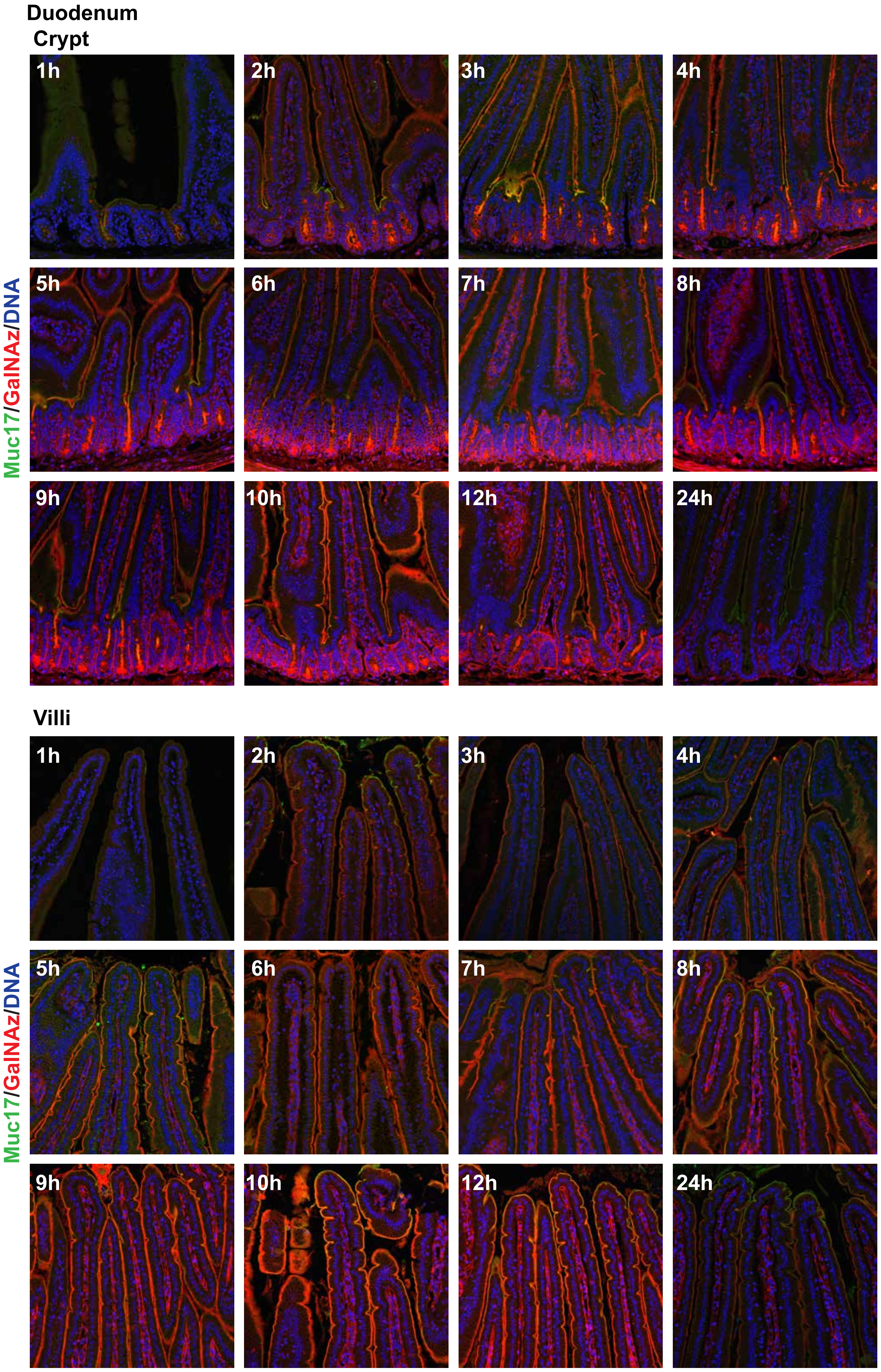

Duodenum
Crypt
Muc17/GalNAz/DNA
1h
2h
3h
4h
5h
6h
7h
8h
9h
10h
12h
24h
Villi
Muc17/GalNAz/DNA
1h
2h
3h
4h
5h
6h
7h
8h
9h
10h
12h
24h

Figure S7

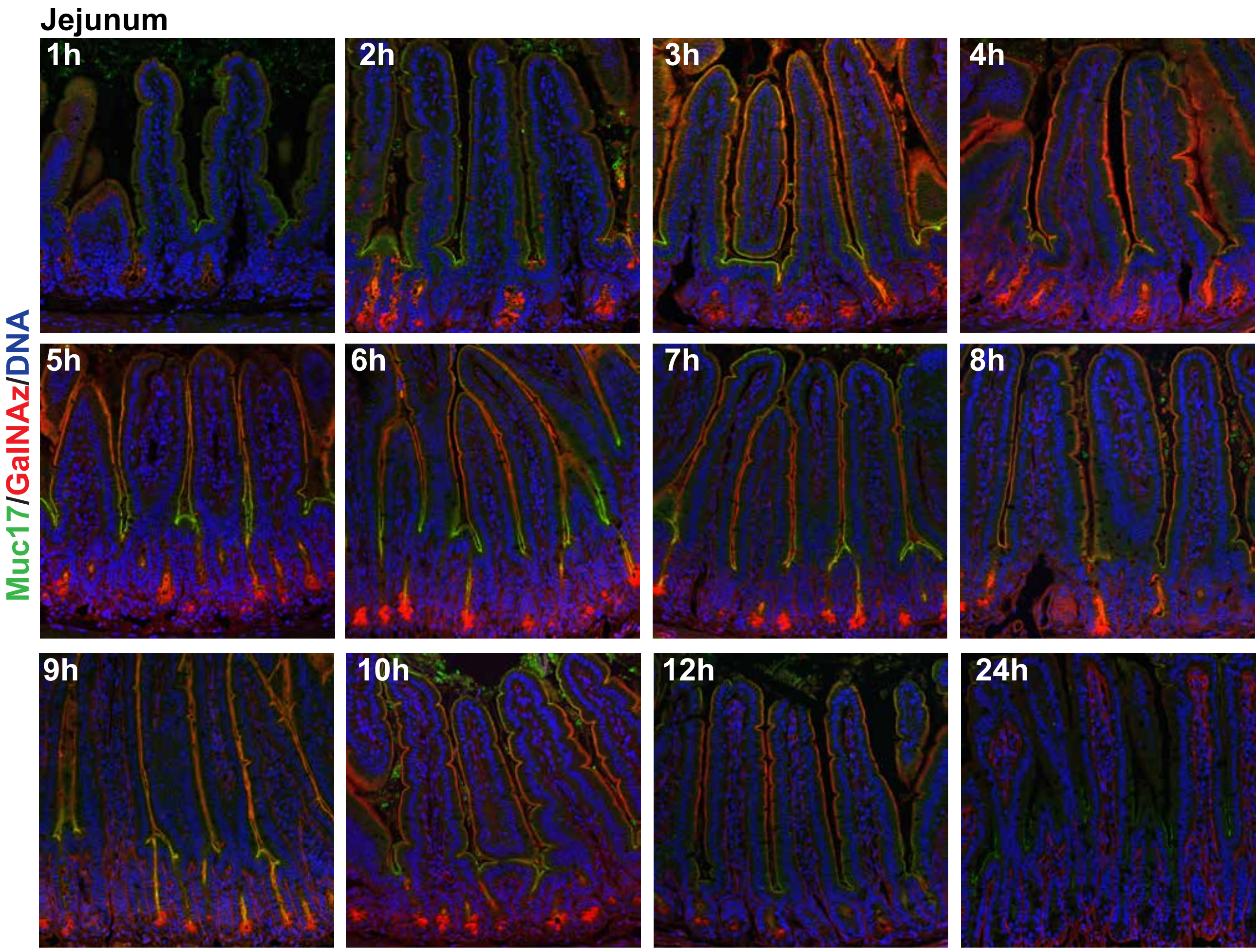

Figure S8

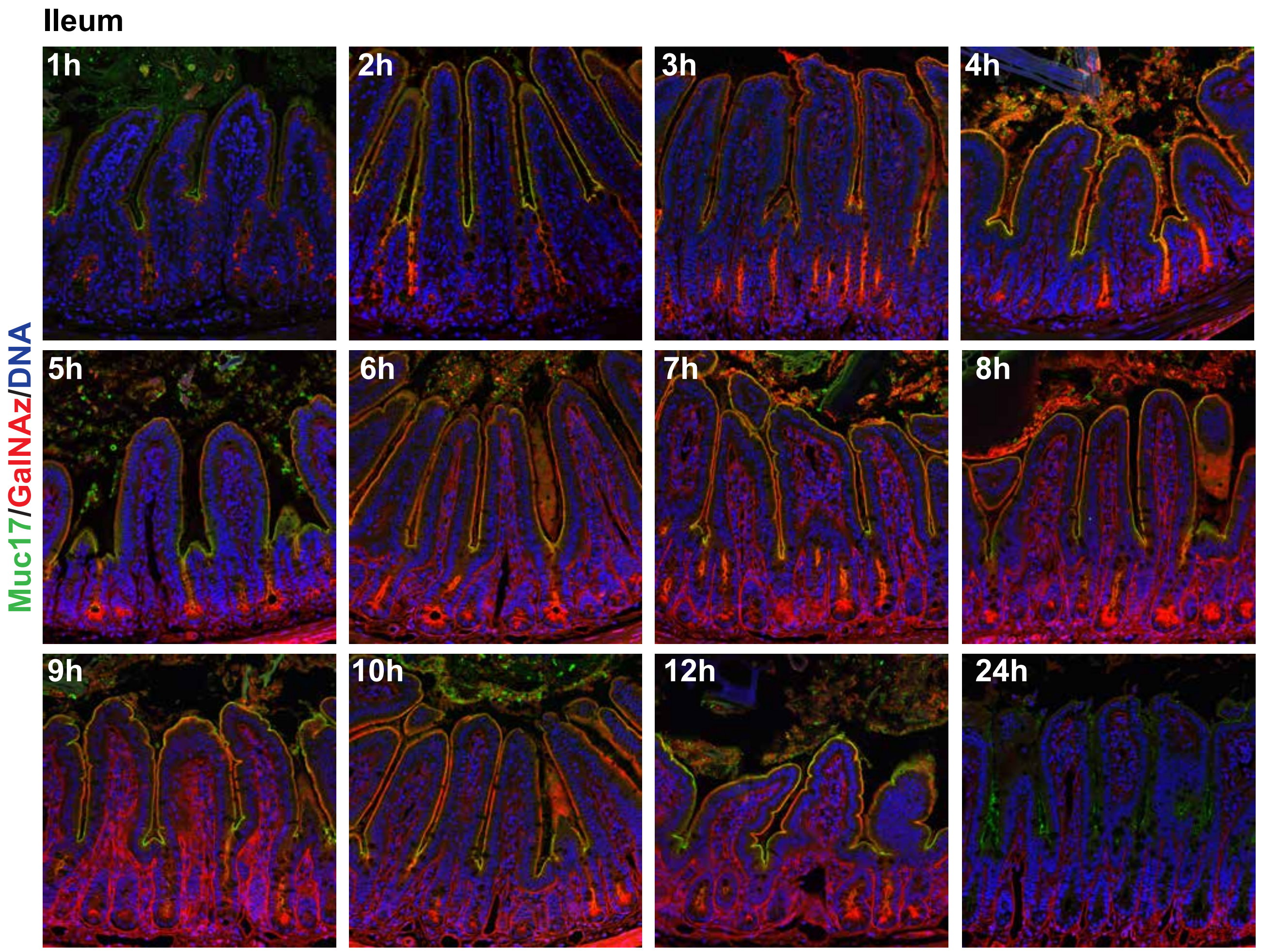

Figure S9

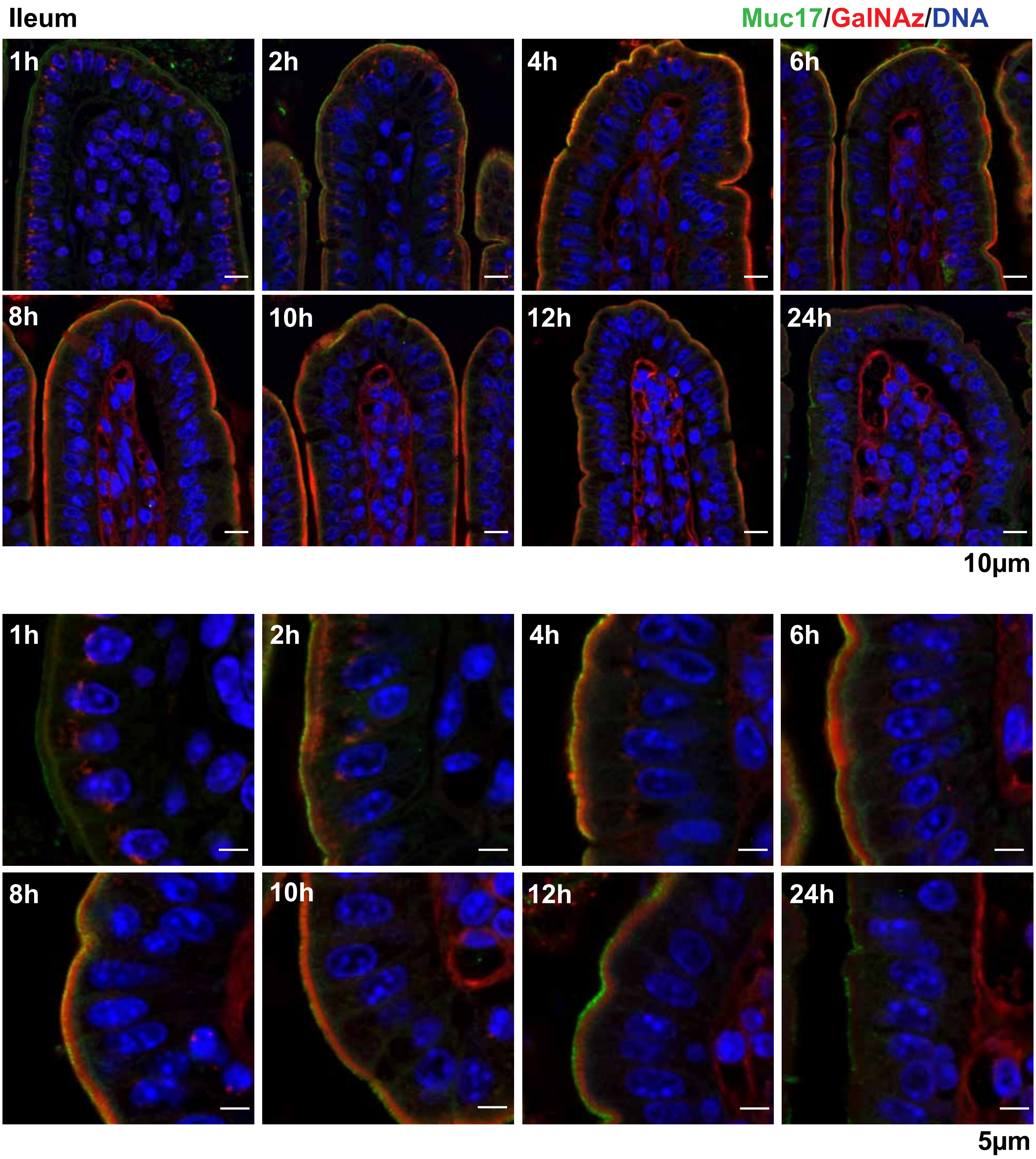
Ileum
Muc17/GalNAz/DNA
1h
2h
4h
6h
8h
10h
12h
24h
10μm
1h
2h
4h
6h
8h
10h
12h
24h
5μm

Figure S10

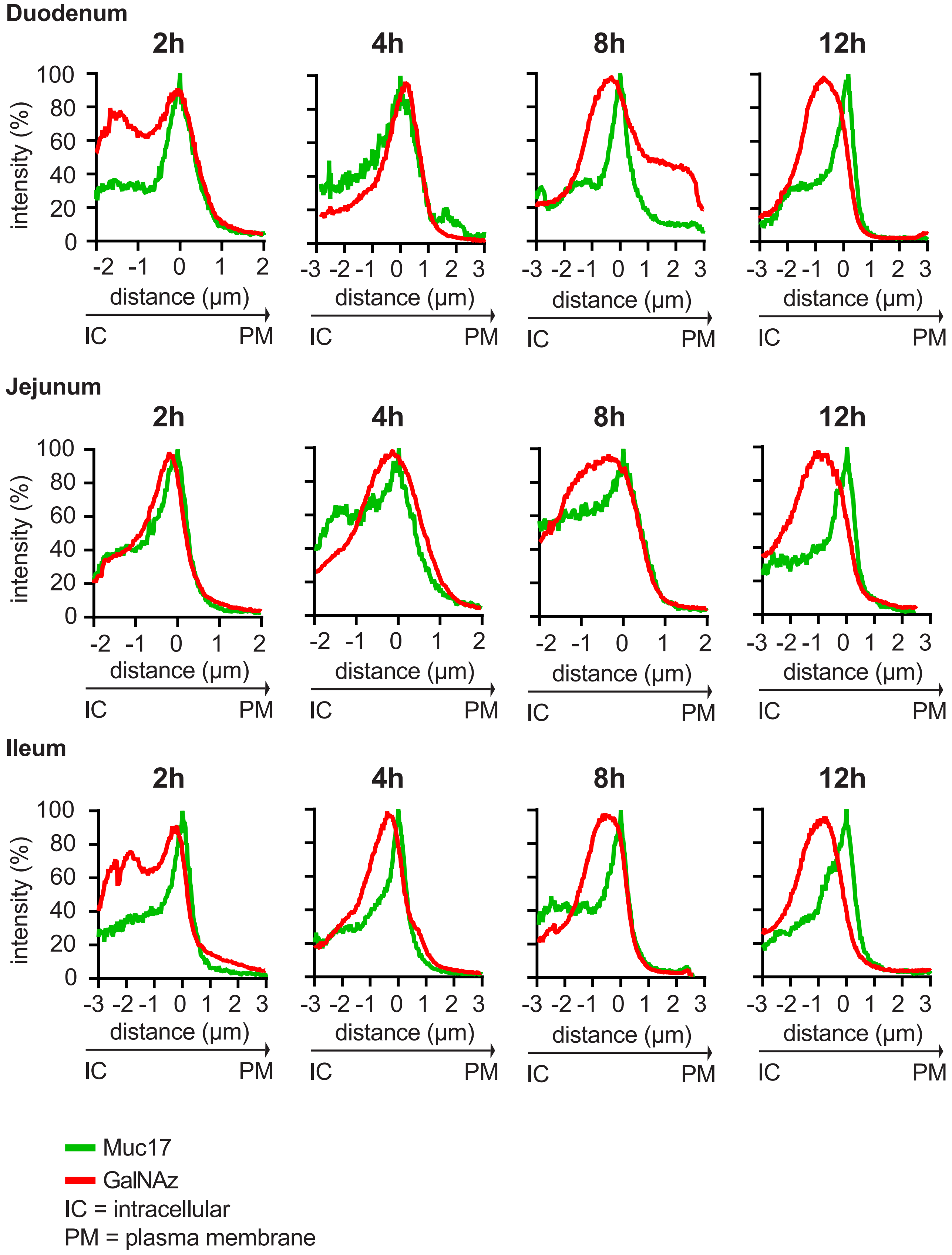
Duodenum
2h
4h
8h
12h
Jejunum
Ileum
intensity (%)
100
80
60
40
20
0
-3 -2 -1 0 1 2 3
distance (µm)
IC
PM
Muc17
GalNAz
IC = intracellular
PM = plasma membrane

Figure S11

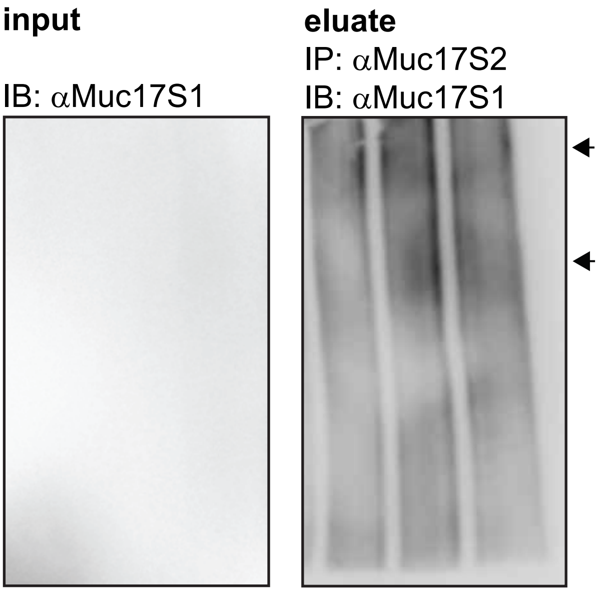

Figure S12

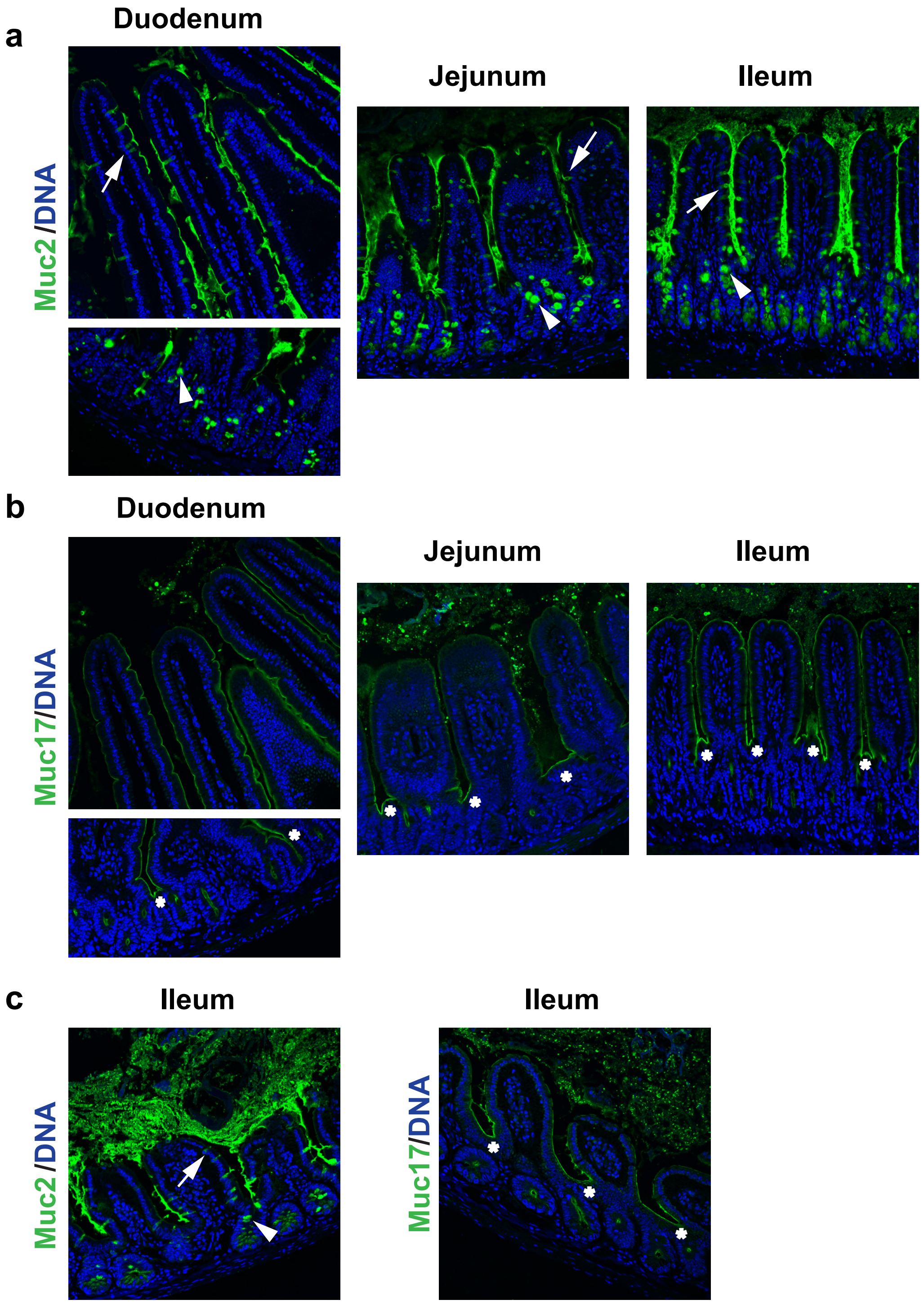
a
Duodenum
Jejunum
Ileum
Muc2/DNA
b
Duodenum
Jejunum
Ileum
Muc17/DNA
c
Ileum
Ileum
Muc2/DNA
Muc17/DNA
